# Supplementary material for: Prevalence of mental distress in adults with and without a history of tuberculosis in an urban Zambian community
Source: Glob Ment Health (Camb). 2023 Nov 28;10:e89. doi: 10.1017/gmh.2023.83 (PMC10755383; doi:10.1017/gmh.2023.83)

Table 1: Comparison of Distribution of SRQ Between Investigation and Control Groups

| **Question** | **N who reported yes** | **% In total sample** | **% In participants with No history of TB** | **% Participants with History of TB** | **% Difference (history of TB – no history of TB)** |
| --- | --- | --- | --- | --- | --- |
| Do you sleep badly | 318 | 9.37 | 9.4 | 10.0 | 0.7 |
| Do you often have headaches? | 693 | 20.0 | 19.9 | 24.2 | 4.3 |
| Do you find it difficult to enjoy daily activities? | 424 | 12.2 | 12.3 | 11.7 | -0.6 |
| Are you unable to play a useful part in life? | 802 | 23.1 | 23.1 | 21.7 | -1.4 |
| Is your daily life suffering? | 512 | 14.7 | 14.5 | 23.3 | 8.8 |

Figure 1: Distribution of SRQ scores between TB survivors and adults with no medical history of TB


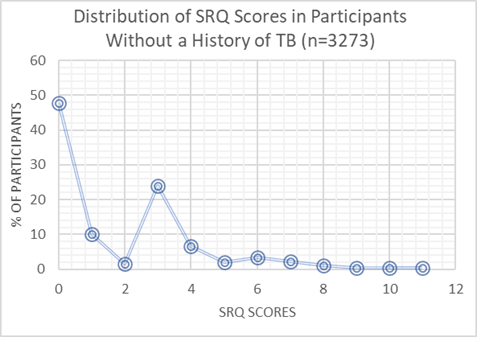

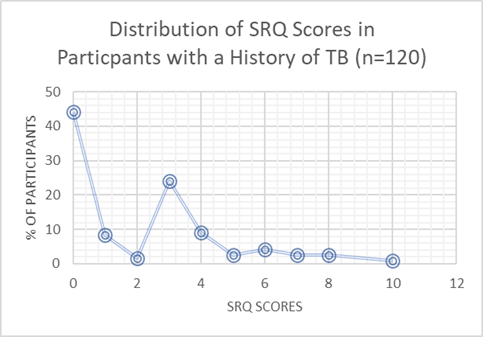

Supplement: Mainga et al. supplementary material [file S2054425123000833sup001.docx]
